# Supraspinal Mechanisms of Spinal Cord Stimulation in Pain Mitigation: A Systematic Review

**DOI:** 10.7759/cureus.86756

**Published:** 2025-06-25

**Authors:** Ayesha Yousaf, Hideaki Yamamoto, Jaden Y Fang, Adam Romman, Aristides P Koutrouvelis, Satoshi Yamamoto

**Affiliations:** 1 Anesthesiology, University of Texas Medical Branch, Galveston, USA; 2 Biological Sciences, University of California San Diego, San Diego, USA

**Keywords:** chronic pain management, neuromodulation, spinal cord stimulation (scs), supraspinal modulation, systematic review

## Abstract

Spinal cord stimulation (SCS) has been established as an effective intervention for chronic pain syndromes, including persistent spinal pain syndrome type 2 (PSPS-T2) and complex regional pain syndrome (CRPS). Nevertheless, the underlying mechanisms responsible for SCS-induced analgesia remain incompletely understood, particularly regarding the supraspinal pathways, which have not been sufficiently clarified. A systematic review was conducted to aggregate evidence concerning the supraspinal influences of SCS on pain modulation. Following an extensive literature search facilitated by a professional librarian and adhering to the Preferred Reporting Items for Systematic Reviews and Meta-Analyses (PRISMA) guidelines, a thorough selection process was executed. Animal studies that investigated the supraspinal circuitry implicated in SCS-induced analgesia were incorporated into the review. Conversely, studies that solely examined spinal effects or lacked appropriate experimental controls were excluded from consideration. Ultimately, four studies fulfilled the inclusion criteria. Collectively, these investigations revealed direct anatomical projections from the dorsal column nuclei (DCN) to the periaqueductal gray (PAG), a reduction in extracellular gamma-aminobutyric acid (GABA) levels within the PAG following SCS, which facilitates disinhibition of descending pain pathways, and heightened activity of OFF-like and serotonergic neurons in the rostral ventromedial medulla (RVM), thereby contributing to antinociceptive outcomes. SCS engages a network of supraspinal structures, particularly the DCN-PAG-RVM axis, to mediate analgesia via neurotransmitter modulation and descending inhibitory mechanisms. These results underscore a complex supraspinal contribution to the efficacy of SCS and advocate for future investigations into optimized neuromodulation protocols that target brainstem circuitry.

## Introduction and background

Spinal cord stimulation (SCS) has been extensively utilized as an interventional modality for chronic pain syndromes, particularly among individuals diagnosed with persistent spinal pain syndrome type 2 (PSPS-T2) and complex regional pain syndrome (CRPS) [1-3]. Formerly known as failed back surgery syndrome (FBSS), PSPS-T2 is the modern term used to describe chronic pain that persists post-spinal surgery, regardless of the surgical procedure or initial pathology [3]. Although post-laminectomy syndrome is often used interchangeably with FBSS, it specifically refers to pain following laminectomy, while PSPS-T2 encompasses pain after any type of spinal surgery [3].

While the effectiveness of SCS in alleviating pain is thoroughly established, the specific mechanisms underlying its supraspinal effects remain inadequately elucidated [4,5]. Current clinical methodologies predominantly emphasize the optimization of stimulation parameters to attain analgesia; however, the more extensive neuromodulatory ramifications of SCS on supraspinal structures necessitate additional investigation [6]. Emerging data imply that SCS influences various supraspinal pathways, including the periaqueductal gray (PAG), rostral ventromedial medulla (RVM), thalamus, and cortical areas associated with pain perception and modulation [7,8]. Functional imaging investigations have revealed altered neural activity within the anterior cingulate cortex (ACC) and insular cortex subsequent to SCS, suggesting a potential involvement in the affective and cognitive aspects of pain processing [9]. Moreover, the thalamocortical loop has been indicated as significant in the analgesic effects induced by SCS, reinforcing the premise that pain modulation transcends spinal cord mechanisms [10].

Despite these observations, a thorough synthesis of the supraspinal mechanisms remains absent, constraining the capacity to refine therapeutic approaches and enhance patient outcomes. The intricacy of pain perception is characterized by complex interactions between ascending and descending pathways, neurotransmitter systems, and cortical processing [11]. The descending pain modulatory system, particularly the PAG-RVM axis, is instrumental in either amplifying or inhibiting nociceptive transmission [12]. The activation of these specific regions via SCS may facilitate the release of endogenous opioids and modulate neurotransmitter systems such as gamma-aminobutyric acid (GABA) and serotonin, thereby contributing to prolonged pain relief [13].

Additionally, recent innovations in neuroimaging and electrophysiological research have yielded insights into the broader neuromodulatory impacts of SCS. Alterations in cortical excitability, connectivity shifts within pain-related networks, and modulation of autonomic responses imply that SCS exerts its effects beyond mere spinal inhibition [14]. Nonetheless, the variability in study designs, patient demographics, and stimulation parameters complicates the interpretation of results, thereby necessitating a systematic methodology to consolidate the existing body of knowledge [15].

This systematic review aspires to critically assess the supraspinal effects of SCS, amalgamating findings from non-human studies. By synthesizing contemporary hypotheses and pinpointing deficiencies in comprehension, this review endeavors to establish a foundation for forthcoming research and clinical advancements in the domain of neuromodulation for pain management [16,17]. An enhanced understanding of these mechanisms may facilitate the development of personalized stimulation protocols, refined patient selection criteria, and innovative therapeutic applications extending beyond the management of chronic pain [18,19].

## Review

Methods

A comprehensive literature search was conducted across several medical and scientific databases, including PubMed, MEDLINE, ScienceDirect, and Google Scholar, to identify animal studies from inception to October 2024. The objective was to review experimental animal studies investigating pain modulation via SCS. Relevant articles were screened and reviewed to ensure thorough coverage. Two independent authors (HY and JF) performed data extraction and article screening based on inclusion criteria. Any discrepancies were resolved by consensus. This protocol was formally registered with the International Platform of Registered Systematic Review and Meta-analysis Protocols (INPLASY) under registration number 202540047. Screening followed Preferred Reporting Items for Systematic Review and Meta-Analyses (PRISMA) guidelines [20].

Search Strategy

Our investigation was carried out in meticulous collaboration with a qualified medical librarian to guarantee an exhaustive and systematic extraction of pertinent studies that elucidated potential mechanisms of pain in relation to the application of SCS in animal models. Subsequent to an initial extensive systematic inquiry, it aimed at identifying relevant literature, as delineated in Table 3 of Appendices.

Study Selection

This systematic review focused on studies that investigated SCS on pain mechanisms in experimental animal models. Specifically, we included studies that applied SCS and investigated its effects on supraspinal structures, including neurotransmitter release, anatomical connectivity, and pain modulation. Studies that incorporated brain structures, such as the PAG or RVM, were considered only if they established a direct anatomical or functional link to spinal components. Spinal cord-related cell types, specifically dorsal horn neurons, interneurons, and glial cells, were excluded from our search. Research that lacked control groups, used non-animal models, or lacked a clear assessment of pain mechanism pathways was excluded. 

Risk of Bias Assessment

Using the SYRCLE’s Risk of Bias tool, which is specifically tailored toward animal research, we methodically evaluated the quality of each study [21]. Adapted from the Cochrane risk of bias framework, this tool investigates potential sources of bias across 10 domains, including randomization, blinding, allocation concealment, and outcome reporting. Each category was rated as “high” or “low” risk of bias. Instead of assigning an overall score, assessments were made based on each criterion. 

Data Collection

The primary objective of data collection was to synthesize findings on how SCS influences pain mechanisms in animal models. We reviewed each study’s authors, publication year, animal species used, type of pain model, outcome measures, intervention protocols, tissue type examined, and key findings. To ensure adherence to structured, transparent reporting, this review was developed using a PRISMA checklist. 

Results

Study Identification and Inclusion

**Figure 1:**
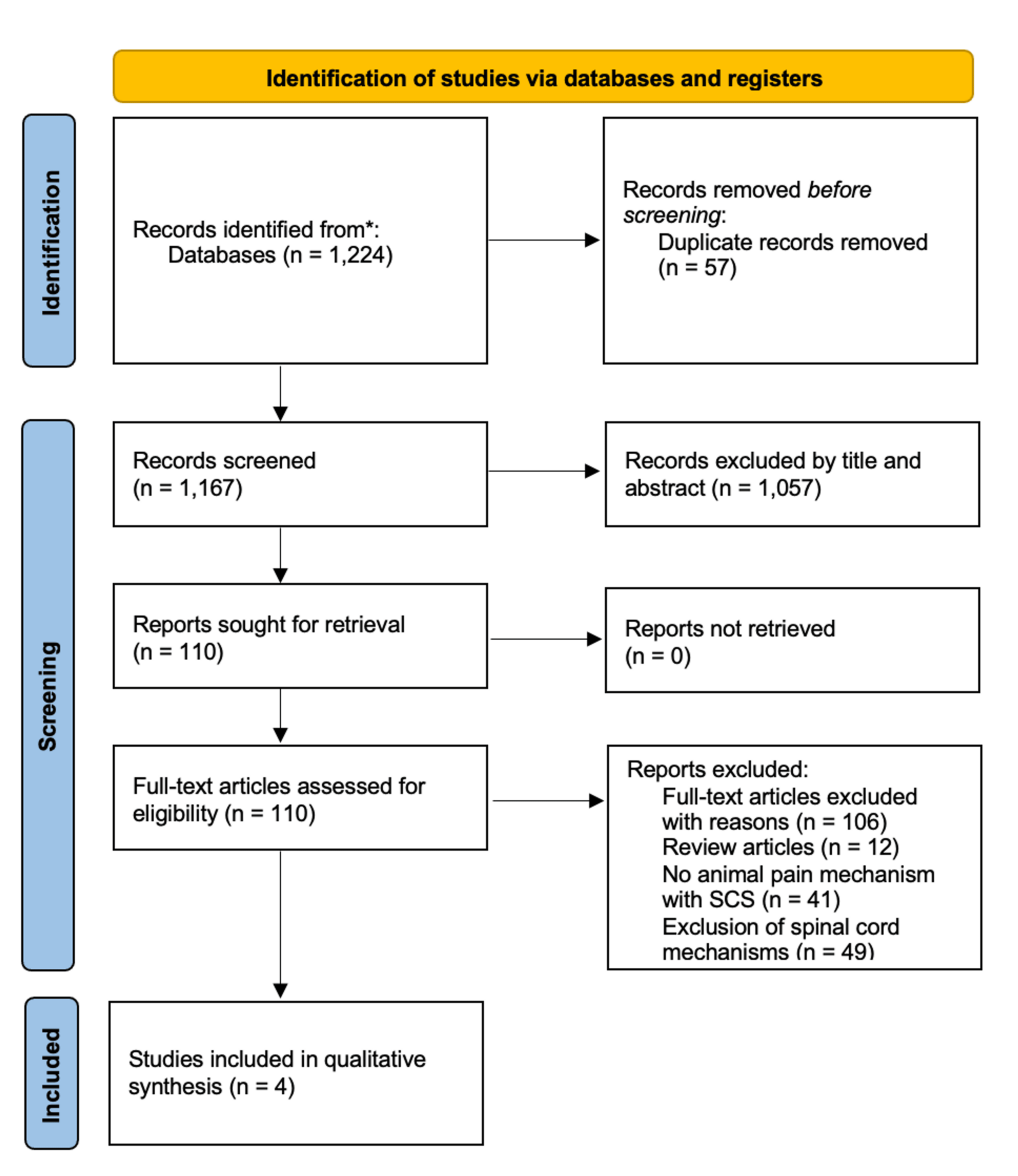
PRISMA flow diagram of the study selection process. *Refer to the Appendices for the study selection strategy. PRISMA, Preferred Reporting Items for Systematic Reviews and Meta-Analyses; SCS, spinal cord stimulation

In our initial research, we identified 1,224 records; however, after removing 57 duplicates, 1,167 records remained for screening. Of the 1,167, 1,057 records, based on title and abstract, were excluded as they were neither relevant to the topic nor adherent to inclusion criteria or used non-experimental designs. Thus, 110 articles remained and were reviewed and screened for eligibility. Studies using human subjects, spinal cord cells, and systematic reviews were excluded. Following full-text evaluation, four articles ultimately met the inclusion criteria and were selected for qualitative synthesis (Figure 1).

A summary of each included study’s authors, publication year, animal species used, type of pain model, outcome measures, intervention protocols, cell type examined, and key findings can be found in Table 1.

**Table 1:** Characteristics of studies with pain modulation moderated by SCS in supraspinal circuits. DCN, dorsal column nuclei; PAG, periaqueductal gray; GABA, gamma-aminobutyric acid; RVM, rostral ventromedial medulla; SCS, spinal cord stimulation; 5-HT, 5-hydroxytryptamine; SNI, spared nerve injury

Characteristics of four studies with pain modulation moderated by SCS in supraspinal circuits						
Author and year	Country	Species	Cell types involved	Study type	Pain model used	Conclusion
Barbaresi et al., 2016 [22]	Italy	Adult rats, Sprague-Dawley	Neurons in DCN and PAG	Anatomical tracing study	N/A	Somatotopically organized projection from DCN to PAG, indicating the structural basis for pain modulation via PAG
Stiller et al., 1995 [23]	Sweden	Male rats, Sprague-Dawley	GABAergic neurons in the PAG; neurons projecting from PAG to nucleus raphe magnus; possibly opioid-sensitive pathways	In vivo neurochemical study	No pain model	SCS-induced reduction in GABA disinhibits PAG neurons that activate descending inhibitory pathways.
Song et al., 2013 [24]	Sweden	Male rats, Wistar	RVM ON-like, OFF-like, neutral and 5-HT-like neurons	In vivo electrophysiology and pharmacological manipulation	SNI model	SCS-induced pain relief is associated with increased RVM-OFF and 5-HT-like cell activity, involving GABAergic transmission
Linderoth et al., 1993 [25]	Sweden	Male rats, Sprague-Dawley	Neurons in the PAG	Experimental/animal model	No direct pain model; baseline neurotransmission measured under SCS	SCS alters neurotransmitter levels in the PAG, suggesting a central mechanism of pain modulation

The overall bias risk for the included studies is summarized in Table 2. Sources of bias across 10 domains, including sequence generation, baseline characteristics, allocation concealment, random housing, blinding of caregivers and/or investigators, random outcome assessment, blinding of outcome assessors, incomplete outcome data, selective outcome reporting, and other potential sources of bias, were reviewed and assessed.

**Table 2:** SYRCLE’s risk of bias assessment for the included studies.

SYRCLE'S risk of assessment				
Domain	Barbaresi [22]	Stiller [23]	Song [24]	Linderoth [25]
Year	2016	1995	2013	1995
Sequence generation	High	High	High	High
Baseline characteristics	Low	Low	Low	Low
Allocation concealment	High	High	High	High
Random housing	High	High	High	High
Blinding of caregivers and/or investigators	High	High	High	High
Random outcome assessment	High	High	High	High
Blinding of outcome assessor	High	High	High	High
Incomplete outcome data	Low	Low	Low	Low
Selective outcome reporting	Low	Low	Low	Low
Other sources of bias	Low	Low	Low	Low

Discussion

This systematic review consolidates four studies from relevant animal models investigating SCS-induced pain modulation. While the Gate Control Theory provides a framework for segmental spinal mechanisms, the selected evidence here highlights the involvement of supraspinal pathways [26]. These include dorsal column nuclei (DCN) projections to the PAG, neurotransmitter modulation within the PAG, and activation of RVM circuits [22-24]. Together, the interactions of these central structures, along with spinal segments, serve as the foundation for mediating SCS-induced analgesia.

While dorsal column projections are primarily associated with fine touch, proprioception, and vibratory sensory input, recent studies indicate that the DCN also receives nociceptive input. Barbaresi et al. (2016) used biotinylated dextran amine (BDA) tracing to demonstrate a direct anatomical link between the DCN and contralateral regions of the PAG. Specifically, the projections from the DCN to the PAG exhibit a somatotopically organized pattern, with the gracile nucleus projecting to the ventrocaudal region of the PAG (associated with tail and hind limb areas) and the cuneate nucleus projecting to the middle and rostral regions of the PAG (corresponding to the forelimb and facial regions). This finding aligns with the phenomenon of somatotopic analgesia observed in studies involving stimulation of the PAG, suggesting that SCS may activate ascending dorsal column pathways that facilitate the activation of PAG neurons by attenuating GABAergic inhibition, consequently resulting in the disinhibition of descending analgesic pathways [22]. Despite these findings, evidence also demonstrates that damage to the DCN does not abolish SCS-induced analgesia [25]. These findings suggest the existence of alternative supraspinal routes activated by SCS, allowing for compensatory modulation.

Neurotransmitter modulation within the PAG highlights the importance of supraspinal pathways in SCS-induced pain modulation. Stiller et al. (1995) found that awake, freely moving rats exhibited decreased extracellular GABA levels in the ventrolateral PAG after repeated SCS sessions. Under conditions of tonic GABAergic inhibition, SCS facilitates the disinhibition of outputs directed toward descending pain-modulating centers, including the nucleus raphe magnus [23]. This attenuation of GABAergic activity consequently activates descending pain modulation pathways, thereby augmenting antinociceptive responses. These findings indicate a differential role of GABAergic mechanisms in the spinal versus supraspinal segments [23]. While SCS leads to a decrease in GABA levels within the PAG, the same investigation revealed an SCS-induced elevation of GABA levels in the dorsal horn, indicating region-specific modulation.

Along with the PAG, the RVM serves as a key relay station in SCS-induced analgesia [24]. Song et al. (2013) found that after spinal nerve injury (SNI), rats exhibited a decrease in OFF-cell activity, which inhibits nociceptive transmission, and an increase in ON-cell activity, which facilitates it. However, following SCS, responding SNI rats demonstrated increased firing of OFF-like and 5-HT-like cells. Moreover, microinjection of the GABAA receptor agonist muscimol resulted in partially reduced anti-hypersensitivity effects of SCS, highlighting the importance of GABA transmission in this region. Together, these findings underscore the significance of DCN-PAG-RVM-spinal circuitry in SCS-induced pain modulation.

This systematic review has a few limitations. We included four studies in this review, limiting the generalizability of the findings. Moreover, the variability in study designs and outcomes made it difficult to directly compare outcomes. Additionally, all studies demonstrated a high risk of bias in certain areas such as random housing, sequence generation, and other domains, which reduced overall confidence in the findings. Direct statistical comparisons were also limited due to the variability in primary measured outcomes, with some studies focusing on neurochemical, electrophysical, or behavioral responses. Finally, this review focused exclusively on preclinical studies; while this approach enabled a detailed mechanistic analysis, the lack of human studies limits the translational applicability of the findings to clinical settings.

Further investigation should explore the significance of the DCN-PAG pathway in SCS-induced analgesia. Specifically, future work should address whether the pathway plays an integral role or represents one among several compensatory anti-nociceptive mechanisms. Additionally, the literature in this review focused on the stimulation of cells and neurotransmitter levels; however, future research should utilize other techniques, such as optogenetics, for a comprehensive understanding of selective GABAergic, serotonergic, and OFF-/ON-target cells in the RVM. Finally, evaluating stimulation parameters, including intensity, amplitude, and waveform, in supraspinal versus segmented spinal circuits can help fill current gaps in research.

## Conclusions

Emerging evidence demonstrates the importance of supraspinal modulation in the mechanisms mediating SCS-induced analgesia. Descending pain inhibition involves key structures, including the PAG and RVM, extending the scope of pain modulation beyond segmental spinal structures. While these studies represent promising strides in the field, there remains a pressing need for further investigation. Continued research aimed at comprehensive analysis and standardized protocols may significantly clarify the roles brainstem structures play in SCS. Further exploration of supraspinal circuits may enhance neuromodulation strategies and support the development of more diverse pain therapies.

## Appendices

**Table 3:** Full search strategy utilizing the Cochrane Central Register of Controlled Trials. *Locates all variations of a root word (e.g., stimulat retrieves stimulate and stimulation), functioning as a truncation symbol. The NEAR/n operator (e.g., NEAR/3) performs proximity searching, identifying terms occurring within n words of each other in any order. Boolean operators (AND, OR) were used to combine search concepts. MeSH descriptors were "exploded," when applicable, to include narrower terms. The final query (line #25) targeted animal studies related to spinal cord stimulation, pain mechanisms, and supraspinal pathways.

ID	Search
#1	MeSH descriptor: [Spinal Cord Stimulation] explode all trees
#2	MeSH descriptor: [Electric Stimulation] explode all trees
#3	MeSH descriptor: [Electric Stimulation Therapy] this term only
#4	((spine or spinal or transspinal or "trans spinal" or column* or epidural or lumbar or "dorsal column" or sacral or coccygeal or "conus medullari" or "conus medullaris" or "conus terminali" or "conus terminalis" or "medulla spinali" or "medulla spinalis" or myelon* or "thoracic cord" or "thoracic cords") NEAR/3 stimulat*)
#5	spinal cord stimulation or "spinal cord stimulations"
#6	(stimulat* NEAR/1 frequency)
#7	((therapy or therapies) NEAR/1 frequency)
#8	(burst NEAR/1 stimulat*)
#9	MeSH descriptor: [Electrodes, Implanted] explode all trees
#10	(electrode* NEAR/1 implant*)
#11	neuromodulat*
#12	#1 OR #2 OR #3 OR #4 OR #5 OR #6 OR #7 OR #8 OR #9 OR #10 OR #11
#13	MeSH descriptor: [Pain] explode all trees
#14	MeSH descriptor: [Pain Management] explode all trees
#15	(pain or pains):ti,ab,kw
#16	MeSH descriptor: [Nociceptors] explode all trees
#17	(nocicept*):ti,ab,kw
#18	#13 OR #14 OR #15 OR #16 OR #17
#19	#12 AND #18
#20	mechanis*
#21	#19 AND #20
#22	MeSH descriptor: [Models, Animal] explode all trees
#23	(animal* or rodent* or rat or rats or mouse or mice or "guinea pig" or "guinea pigs" or hamster* or rabbit* or cat or cats or dog or dogs or cattle or cow or cows or horse or horses or donkey* or mule or mules or monkey* or "nonhuman primate" or "non-human primate" or "nonhuman primates" or "non-human primates" or chimpanzee* or sheep or swine or pig or pigs or chicken or chick or chicks):ti,ab,kw
#24	#22 OR #23
#25	#21 AND #24
